# The Effect of Motor Learning of Serial Reaction Time Task (SRTT) Through Action Observation on Mu Rhythm and Improvement of Behavior Abilities

**DOI:** 10.4021/jocmr727w

**Published:** 2012-03-23

**Authors:** Sang-Yeol Lee, Sung-Soo Bae, Jin-Tae Han, Seung-Deuk Byun, Jong-Sung Chang

**Affiliations:** a Department of phyical therapy, Kyoungsung University, Republic of Korea; b Department of Physical Therapy, Daegu University, Gyeongsan, Republic of Korea; c Department of Physical Therapy, Kyungsung University, Pusan, Republic of Korea; d Department of Physical Medicine and Rehabilitation, Daegu Fatima Hospital, Daegu, Republic of Korea; e Department of Physical Therapy, Daegu Haany University

## Abstract

**Background:**

The aim of this study was investigate whether an action observation would have an effect on the action that requires task understanding in humans.

**Methods:**

Participants who met the criteria for this study (n = 36). To evaluate the performance, reaction time and performance accuracy, the stimulus scheduling software was employed. For the electroencephalogram, the equipment QEEG-8 was used.

**Results:**

Concerning the reaction time of the groups of different learning methods a significant difference was found after the learning among the three groups. Regarding the accuracy among the groups of different learning methods, significant difference was found in the action performance accuracy among the three groups. The relative mu power during the SRTT implementation was compared in the CZ, C3 and C4 regions before, during, and after the learning for each group. In the CZ and C4 region, a significant difference was found in the action observation group. In the C3 and C4 region a significant difference was found in the actual practice group.

**Conclusions:**

The result suggests that imitation and learning are involved even in the action that requires task understanding in humans.

**Keywords:**

Accuracy; Mirror neuron system; Mu Rhythm; Reaction time; SRTT

## Introduction

Motor learning is the human ability to maintain flexibility to the environment in learning and performing motor skills [1]. Motor learning is a comprehensive concept that includes the sensory processing procedure, motor control, and motor skill learning and it also refers to the ability to perform and memorize learned skill under variety of conditions [2]. It was known that traditional motor learning can be obtained by means of repeated movement. In fact, however, most of the motor functions include both physical and cognitive factors. Recently, studies have actively been conducted showing that motor skill can be obtained through mental practice. In the case of the traditional movement method through direct practice, motor learning is difficult if there is limitation in physical movement. For example, initial motor learning is impossible for stroke patients in their initial stage because of the limitation in movement. The indirect practice methods to supplement such a drawback include mental practice through motor imagery and action observation using the visual and auditory senses [3, 4]. Studies are actively carried out about the effect of mental practice by means of motor imagery among the motor learning methods through indirect practice. However, disadvantages such as lowered level of concentration during the learning should be supplemented. For that, action observation using the visual and auditory senses have been actively studied in recent times [3].

Action observation is the method to understand, choose and imitate the form and motion of an action by observing other's action. [1]. Initially, action observation was the research theme of social psychology about imitation [2], and it is nowadays highlighted as a method for cognitive intervention to supplement the limitation of motor imagery [3]. Recent studies using the brain image during action observation reported that the brain area involved in actual practice was activated during action observation [3, 5], and the motor evoked potential of the corresponding area was also elevated [6, 7]. These results mean that action observation can produce the same neuromuscular response with that of actual practice. When the dynamic imaging of finger movement for SRTT was shown to adults, the brain response that was similar to that by actual practice was found even by the observation through dynamic imaging [3]. This was reported as the result of the action of the brain mirror neuron [3, 8]. The activation of the mirror neuron suggests that action observation for a teleological and well-trained action can help to form the appropriate, coordinated patterns of the action and learn the action by activating the brain area involved in the same action [9].

Perry and Bentin [10] studied the effect of oneness in the objective and grip shape and reported that mu rhythm was repressed the most and the mirror neuron in the mirror neuron was activated the most. This result suggests that there is a high correlation between the mirror neuron activation and the mu rhythm repression that take place during action observation.

The mu rhythm, an electroencephalogram, is in the range of alpha wave, but it has difference features from alpha wave. Although alpha wave is relatively regular, the mu rhythm looks like a bow and is often asymmetric and asynchronous [11]. While the amplitude of alpha wave is 30 - 50 μV, the amplitude of the mu rhythm is lower than that. While alpha wave appears in all the brain areas, mu rhythm appears usually around the central sulcus (CZ, C3 and C4) in general [12]. Many recent studies showed that action observation is involved in motor learning as it greatly affects the mirror neuron activation and mu rhythm repression.

However, even though many studies and analyses have been carried out on the action observation of the movement with simple objectives, the effect of the performance observation of the task where the response to stimulus is required, such as SRTT, on the task accuracy and reaction time has been little studied. In this study, we investigate the mu rhythm and the mirror neuron system activation that appears during the action observation, motor imagery, and actual practice of the task that requires understanding of movement, such as SRTT, and studied the effect of the individual motor learning methods on the movement accuracy and reaction time.

## Methods

This study was conducted with a total of 36 healthy adults at the age of 20's including 12 subjects in the actual practice group, 12 subjects in the action observation group, and 12 subjects in the motor imagery group. There was no significant difference in the age, weight and height among the three groups (P > 0.05).

All the subjects sat in front the desk on which a computer is laid. The height of the chair was controlled so that the elbow joint angle could be about 90° when the right upper limb was placed on the desk. The subjects were asked to perform the task as accurately and quickly as possible following the visuoauditory signals. While performing the task, the subjects performed the consecutive reaction-time tasks with the minimum finger movement, fixing the elbow joint and the wrist joint.

**Figure 1:**
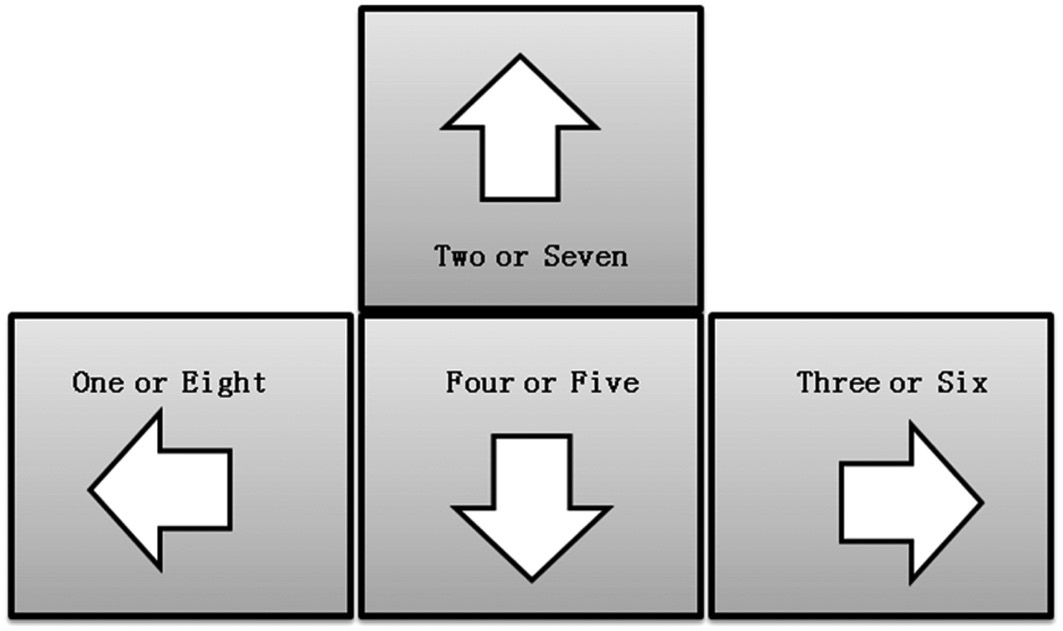
Method of reaction.

The SRTT for this study was to press the corresponding key on the keyboard with the designated figure when 40 consecutive signals were provided. The 40 signals included "one" to "eight" that were repeated for five times each. The subjects were asked to press the left-arrow key with the index finger for "one" and "eight," the up-arrow key with the ring finger for "two" and "seven," and the down-arrow key with the middle finger for "four" and "five" (Fig. 1). The forty consecutive signals composed of five-time repetition of eight signals constituted one block which was implemented for one-time practice. When responding the forty consecutive signals, the next signal follows in one-second interval regardless of the key-pressing by the subjects in response to the consecutive signals. The tasks with the same order were assigned to the subjects. The visuoauditary signals were prepared and provided in the form of dynamic imaging file including the letters and audio files of "one" to "eight." The subjects were asked to perform the task according to the signals. All the subjects wore an earphone while performing the task.

Through the one-time repetition performance in the method described above, we measured the reaction time, accuracy and mu rhythm before learning. The subjects in the actual practice group learned the movement by means of actual practice of three times, while the subjects in the action observation group learned it for three times through the dynamic imaging (visual and auditory) of the performance by the third person who was well-trained . Additionally, the subjects in the motor imagery group learned the movement only by using the visuoauditary file used in SRTT.

To evaluate the reaction time and accuracy of SRTT, we employed the stimulus scheduling software (LAXTHA, Korea). While all the subjects were performing the task, the performance reaction time and the performance accuracy were recorded in all the intervals.

The electroencephalogram was measured using QEEG-8 (LXE3208, LAXTHA Inc., Korea). The electroencephalogram sampling rate of the measured subjects was 256 Hz, filtered in the range of 0.5 - 50 Hz, and the data was saved in a computer by the 12-bit AD conversion. The electroencephalogram was measured in three regions on the head surface using the monopoly system. The electrode was attached using the international 10 - 20 system. To measure the mu rhythm that appears during the motor performance of hand, action observation and motor imagery in this study, three channels, Cz, C3 and C4, were attached, and the reference electrode and ground electrode were attached to the styloid process on the right and left sides.

In this study, to quantify the generation of mu rhythm in the electroencephalogram which was known to be closely related to the mirror neuron activity, the ratio of mu rhythm to the total wave was calculated and referred as relative mu power.

Mu rhythm(μ)/(θ wave + α wave + β wave + γ wave) = (7 - 11 Hz)/( 4 - 50 Hz)

To investigate the change in the behavior in each group before and after the learning, one-way ANOVA and an independent t-test were performed. Repeated ANOVA was used to compare the relative mu power before, during and after the learning in each group.

This study was approved by the Institutional Review Board (IRB) and the approval number is DFH09OT055.

## Results

### Behaviour change after practice

With respect to the reaction time among the groups of different learning methods, no significant difference was found in the reaction time among the group before the learning (P > 0.05), where the reaction time after the learning showed significant differences among the three groups (P < 0.05). The post-hoc test to examine the reaction time difference among the groups after the learning, the reaction time was significantly longer in the motor imagery group (Table 1) (P < 0.05). Regarding the action performance accuracy among the groups of different learning methods, no significant difference was found in the action performance accuracy among the group before the learning (P > 0.05), where the action performance accuracy after the learning showed significant differences among the three groups (P < 0.05). The post-hoc test to examine the action performance accuracy difference among the groups after the learning, the performance accuracy was significantly lower in the motor imagery group (Table 1) (P < 0.05).

**Table 1:** Comparison of Reaction Time and Accuracy

		AO (n = 12)	AP (n = 12)	MI (n = 12)	F	P
RT (msec)	Pre	0.69 ± 0.04	0.69 ± 0.54	0.67 ± 0.58	0.69	0.50
	Post	0.61 ± 0.03	0.61 ± 0.05	0.65 ± 0.05	3.47	0.04*
AC (%)	Pre	68.61 ± 12.06	65.46 ± 12.26	72.96 ± 14.51	1.00	0.37
	Post	86.95 ± 5.74	89.16 ± 8.60	80.18 ± 10.47	3.63	0.03*

mean ± SD; RT: Reaction time; AC: Accuracy; AO: Action observation; AP: Actually practice; MI: Motor imagery;*P < 0.05.

### Mu rhythm change after practice

The relative mu power was compared for the SRTT performance before, during and after the learning in the CZ, C3 and C4 regions in each group. The result showed that there were significant differences in the action observation group and the actual practice group in the CZ region (P < 0.05), while not significant difference was found in the motor imagery group. In the C3 region, a significant reduction was found only in the actual practice group, while a little decreasing tendency was found in the action observation group. In the C4 region, a significant difference was found in the action observation group and the actual practice group (P < 0.05), while no significant difference was found in the motor imagery group (Table 2).

**Table 2:** Comparison of Relative Mu Power Within Intervention Period on Each Group at CZ, C3 and C4 Area

Area	Group	Pre	Mid	Post	F	P
CZ	AO	0.21 ± 0.06	0.18 ± 0.04	0.13 ± 0.05	12.39	0.00*
	AP	0.22 ± 0.08	0.18 ± 0.04	0.13 ± 0.05	2.71	0.08
	MI	0.24 ± 0.06	0.23 ± 0.07	0.22 ± 0.06	0.353	0.70
C3	AO	0.18 ± 0.05	0.17 ± 0.04	0.14 ± 0.04	2.15	0.14
	AP	0.21 ± 0.07	0.18 ± 0.03	0.16 ± 0.061	9.48	0.00*
	MI	0.21 ± 0.03	0.22 ± 0.04	0.20 ± 0.06	0.56	0.57
C4	AO	0.20 ± 0.02	0.16 ± 0.04	0.13 ± 0.04	20.55	0.00*
	AP	0.21 ± 0.08	0.17 ± 0.04	0.15 ± 0.04	11.71	0.00*
	MI	0.24 ± 0.05	0.22 ± 0.07	0.22 ± 0.03	1.35	0.27

*P < 0.05.

## Discussion

Various methods are applied for motor learning and each method has unique characteristics. Learning through actual practice, which is direct motor learning, is effective, but limited by time and space. Although the learning by means of motor imagery, one of the motor learning methods, is less limited by time and space, the learning ability is not maximized in the cases when understanding of the task performance is insufficient and the ability, control and concentration to remind the task performance scene are lacking [13]. However, the motor learning through action observation, which has drawn attention of many researchers recently, is known as the method that helps to better remind the task performance scene specifically than the learning through motor imagery and supplement control and concentration [3]. In this study, to exclude the effect of audiovisual stimulation, all the audiovisual stimulations were provided also to the motor imagery group except the action by the third person.

In this study, we measured the mu rhythm of 7 - 11Hz waveform that is characterized by its repression during the mirror neuron activation in learning process to analyze the learning process status and the degree of concentration. The result showed that the mu rhythm became repressed as the learning proceeded in the CZ, C3 and C4 regions in the actual practice group and the action observation group. This result indicates that accurate learning took place as the mirror neuron was activated in the action observation group and the actual practice group.

The accuracy and reaction time support the result. The accuracy and reaction time were measured in this study for each of the learning methods before, during and after the learning. The result showed that the accuracy was significantly increased after the learning in the actual practice group and the action observation group, and the reaction time was significantly reduced in those groups. This result might have been caused by the repression of the mu rhythm which was known to play the identical functional role with that of the mirror neuron. In the conventional studies where the mu rhythm was observed when observing the movement of a person and a robot, significant repression was found in the C3 and C4 regions, which indicated that the first-order movement region can be activated when observing an objective-oriented movement [10, 13]. In our study also, the result similar to that of previous studies was found. The result suggests that the learning mechanism that involves understanding and imitating of task by mirror neuron system activation takes place not only in the observation of simple actions but also in the performance of task that requires action responding to stimulus such as SRTT.

The result suggests that understanding of task by mirror neuron system activation and learning through imitation take place not only in the observation of simple actions but also in the performance of task that requires action responding to stimulus such as SRTT. It was known that imitation and learning are involved even in the action that requires task understanding in humans.
